# Standardizing estimates of the *Plasmodium falciparum *parasite rate

**DOI:** 10.1186/1475-2875-6-131

**Published:** 2007-09-25

**Authors:** David L Smith, Carlos A Guerra, Robert W Snow, Simon I Hay

**Affiliations:** 1 Fogarty International Center, National Institutes of Health, Building 16, 16 Center Drive, Bethesda, Maryland 20892, USA; 2 Spatial Ecology and Epidemiology Group, Tinbergen Building, Department of Zoology, University of Oxford, South Parks Road, Oxford OX1 3PS, UK; 3 Malaria Public Health & Epidemiology Group, Centre for Geographic Medicine, KEMRI-Wellcome Trust-Collaborative Programme, Kenyatta National Hospital Grounds, PO Box 43640-00100 Nairobi, Kenya; 4 Centre for Tropical Medicine, John Radcliffe Hospital, University of Oxford, Oxford, OX3 9DS, UK; 5 Department of Zoology & Emerging Pathogens Institute, University of Florida, 223 Bartram Hall, PO Box 118525 Gainesville FL, USA

## Abstract

**Background:**

The *Plasmodium falciparum *parasite rate (PfPR) is a commonly reported index of malaria transmission intensity. PfPR rises after birth to a plateau before declining in older children and adults. Studies of populations with different age ranges generally report average PfPR, so age is an important source of heterogeneity in reported PfPR data. This confounds simple comparisons of PfPR surveys conducted at different times or places.

**Methods:**

Several algorithms for standardizing PfPR were developed using 21 studies that stratify in detail PfPR by age. An additional 121 studies were found that recorded PfPR from the same population over at least two different age ranges; these paired estimates were used to evaluate these algorithms. The best algorithm was judged to be the one that described most of the variance when converting the PfPR pairs from one age-range to another.

**Results:**

The analysis suggests that the relationship between PfPR and age is predictable across the observed range of malaria endemicity. PfPR reaches a peak after about two years and remains fairly constant in older children until age ten before declining throughout adolescence and adulthood. The PfPR pairs were poorly correlated; using one to predict the other would explain only 5% of the total variance. By contrast, the PfPR predicted by the best algorithm explained 72% of the variance.

**Conclusion:**

The PfPR in older children is useful for standardization because it has good biological, epidemiological and statistical properties. It is also historically consistent with the classical categories of hypoendemic, mesoendemic and hyperendemic malaria. This algorithm provides a reliable method for standardizing PfPR for the purposes of comparing studies and mapping malaria endemicity. The scripts for doing so are freely available to all.

## Background

The intensity of malaria transmission by its anopheline vectors varies enormously [1], and this affects most quantitative aspects of malaria epidemiology [2]. A commonly used index of malaria transmission intensity is the *Plasmodium falciparum *parasite rate (PfPR), the proportion of the population found to carry asexual blood-stage parasites. In mathematical models, PfPR is related to the entomological inoculation rate (EIR), the number of bites on a person by sporozoite positive vectors, at the steady state [3,4]. The notion of a steady state has limited application for PfPR, however, because PfPR follows a well-established pattern as a function of age and transmission intensity; PfPR rises during infancy and early childhood [5,6], settles to a plateau in older children, and declines in adolescents and adults as malaria immunity develops [7-10]. This pattern has been known for decades [11,12], but there are no established standards for reporting PfPR, so thousands of studies have reported crude PfPR, without stratifying by age [13]. As a result, the different age-ranges over which studies have reported PfPR make it difficult to compare prevalence estimates at different times and places. Assemblies of PfPR data aimed at describing the current global distribution of malaria endemicity [13], therefore, need a mechanism to standardize these data to a consistent age-grouping in order to be meaningfully summarized.

Mathematical models with slowly acquired immunity qualitatively reproduce the empirical relationships between age and PfPR [14-17]. The models suggest that EIR (or the force of infection) determines both the rate that PfPR rises in children and the level of the plateau, so either measure would provide a reliable index of transmission intensity. The PfPR in children is correlated with EIR [4], and the PfPR in children aged 2–10 has provided a basis for the classical categorical measures of malaria transmission: hypoendemic (<10%), mesoendemic (10–50%), and hyperendemic (50–75%) [18]. Holoendemicity refers to PfPR >75% in those less than 12 months old [18].

The variable way of reporting PfPR also limits the number of studies where PfPR can be used to index transmission intensity, which poses a particular problem for recent approaches aimed at using empirical PfPR data to model the spatial distributions of malaria transmission intensity at regional [19-21] or potentially at global scales [22]. For example, maps of transmission intensity in Africa are often based on studies of PfPR that include only children [23,21,25]. The evidence base for mapping malaria, the geographical extent and coverage of places where PfPR has been measured directly, could all be greatly expanded if it were possible to standardize crude PfPR in studies that include adults. The consistent pattern apparent in age-stratified PfPR data suggests that it is amenable to mathematical or statistical modeling. A set of candidate algorithms was derived and evaluated in an attempt to provide a single evidence-based method for converting crude estimates of PfPR to a standard age range for improving future global comparisons of malaria risk using existing extensive PfPR data [13].

## Methods

### Experimental approach and data

This analysis has the distinctive purpose of developing an algorithm to age-standardize PfPR data or, in other words, the purpose is to find a function, *F*, that transforms an estimate of PfPR over any age range *[x,y)*, into a PfPR over a standard age range *[L,U)*, i.e. *F: PR[x,y)*→*PR[L,U)*. This principle was achieved for a number of candidate statistical and biological deterministic models developed from 21 studies that report very detailed PfPR by age (the training set). The skill of the models was then evaluated by inter-conversion of 121 pairs of crude PfPR estimates, where both were taken from the same population but aggregated over different age ranges (the testing set).

The data selection criteria and summary details are given in Additional File 1 and Additional File 2 for the training and testing sets, respectively. Briefly, the data spanned all potential *P. falciparum *transmission intensity categories [18]. The algorithms developed from the training set were validated against the 121 pairs of the testing dataset by comparing the conversions from one age group to the other, usually from adults to children, and *vice versa*. A selection was made of the best performing algorithm using the goodness of fit, measured as the proportion of the variance explained, and defined as the ratio of the variance in residual error divided by the error in the observed PfPR subtracted from one.

The algorithms all differ from each other in significant ways. The linear regression algorithm was based only on the testing set. The other algorithms were based on only the training set, and did not use the testing set for their development. The linear regression algorithm does not predict a relationship between age and PfPR. The interpolation algorithm predicts PfPR in the testing set based on direct interpolation of the training set data; it does not predict an age-PfPR relationship. The two parametric algorithms were fitted to the training set, but they were used only for prediction on the testing set, so parsimony is not an important concern. The most important measure of performance is their skill – a more complicated model that did better at standardizing PfPR would be preferred regardless of the level of complexity, as long as the algorithm had not been fitted to the testing set. Since the linear regression algorithm is a statistical description of the testing set, it sets a standard for judging the skill of the other algorithms. The predictions based on linear regression of the testing set, on the other hand, do raise important issues of parsimony. Because the statistical analysis was one of the candidate algorithms, the analysis was repeated on a sub-sample of the testing set: two-thirds of the data pairs were used for fitting and one-third for validation. Based on these rules, one algorithm was selected as the most appropriate evidence-based method to age-standardize PfPR for future comparisons between studies reporting parasite prevalence across different age ranges.

### Statistical candidate algorithms

#### Linear regression

Linear regression was used to describe the relationship between the pairs of PfPR estimates from the testing set. It differs from the other algorithms in that it is based on a statistical analysis of the testing set; it does not predict a relationship between PfPR and age, and it does not rely on the training set. Let PR_1 _and PR_2 _denote the two estimates made on the intervals [L_1_, U_1_) and [L_2_, U_2_), respectively. The full regression included the age limits: (i.e. PR_1 _= c_0 _PR_2 _+ c_1 _L_1 _+ c_2 _U_1 _+ c_3 _L_2 _+ c_4 _U_2 _+ ε). This formula sometimes returns values for predicted PR_1 _that are outside the interval (0,1), so the analysis was repeated as non-linear regression where the points outside this range were forced to be either 0 or 1. The regression analysis included each pair twice; each member was both PR_1 _once and PR_2 _once. Linear regression of the full testing set provided a standard for the other algorithms, and the predicted values from the linear regression were also evaluated as a potential algorithm.

#### Interpolation

A general class of interpolation algorithms was based on the training set: (i) given the PfPR from a focal study, *PR*_*f*_*[x,y)*, compute the PfPR for the training sets over the same interval, *PR*_*i*_*[x,y), i = 1...21*; (ii) let *W*_*i *_= | *PR*_*f*_*[x,y) - PR*_*i*_*[x,y) *| ^-*z*^, and let *ω*_*i *_= *W*_*i*_/∑_*i*_*W*_*i *_denote the weight of the i^th ^PfPR estimate and (iii) the standardized PfPR is then ∑_*i*_*ω*_*i*_*PR*_*i *_*[L,U)*. Linear interpolation was also considered and was very similar to the general interpolation algorithm with *z = *6.

#### Pull & Grab-based Algorithm

Let *P(A) *denote the true prevalence and *F(A) *the sensitivity of the microscopy, a standard method for estimating PfPR, as a function of age. The function *F(A) *was motivated by the notion that sensitivity declines with age as blood-stage immunity reduces parasite densities to a point where they are often below the detection thresholds of microscopy [26,27]. Therefore, the apparent PfPR, the one that a study would find using microscopy, is *p(A) = P(A) F(A)*. Crude PfPR estimates also depend on the age-distribution of the sampled population, *S(A)*; when PfPR is reported crudely, the age-distribution is generally not reported, so it must also be inferred. Given *p(A) *and *S(A)*, an estimate of crude PfPR would be *PR*_*f*_*[x,y) *= ∫_*x*_^*y*^*p(A) S(A) dA*/∫_*x*_^*y*^*S(A) dA*. The standardized estimate would be *PR*_*f*_*[L,U) *= ∫_*L*_^*U*^*p(A) S(A) dA*/∫_*L*_^*U*^*S(A) dA*.

**Figure 1:**
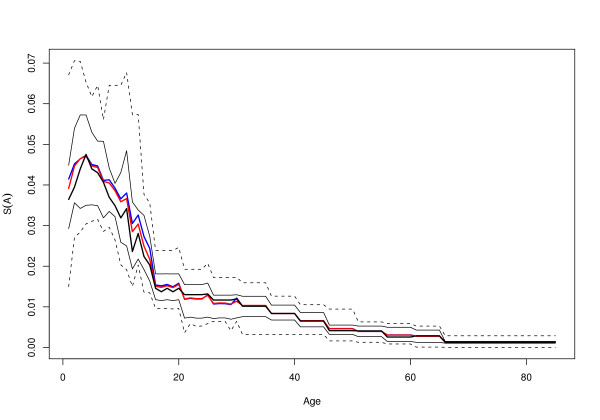
The average distribution of sample sizes, S(A), from 21 studies that report PfPR by age, A. The lines show, by age, the mean (blue), the trimmed mean (red, with 10% of extreme values eliminated), the median (thick black), the interquartile range (thin black), and the 5^th ^and 95^th ^quantiles (dashed lines).

The estimate of *S(A) *was based on the age-distributions in the training set (Figure 1). Each of the 21 studies reports the number of people sampled by age or by age class. Typically, ages were binned by year through childhood, then by 5-year age classes up to age 65, and finally the elderly, which was arbitrarily truncated at age 85. When a study binned several age classes, the observations for each year were proportional to the total observed for that class. For example, the sample for 27 year olds was counted as 1/5 of the total observations for 25–30 year olds. In sum, to get an estimate of *S(A)*: (i) for each study, the proportion of all people sampled that belonged to each one-year age class was computed and (ii) the proportion for each age class was the average for all the studies, where each study was weighted equally.

To generate p(A), the curves P(A) and F(A) were generated separately. P(A) was based on Pull & Grab's equations [28], which were motivated by the Ross model [29] and previous work by Muench [30]. The change in PfPR with age is described by the equation:

dP/dA = h (1-P) - r P;

where *h *denotes the force of infection (i.e., the "happenings" rate), and *r *is the rate at which infections clear. When *P(0) = 1*, this equation has the solution:

*P(A) = P' (1 - e*^-*bA *^*)*.

Here, *P' = h/(h+r) *is the PfPR at equilibrium, and *b = h+r *describes the rate at which PfPR approaches equilibrium.

A three-parameter function was used for *F(A)*: 1 - s [1 - min (1, e^-*c*(*A*-*α*)^) ]. Beginning at age a, this function declines from 1, to 1-s; the parameter c describes the decline from 1 to 1-s as a function of age. Conceptually, this function can be thought of as the decline in the probability of detecting an active infection, although the real reason for the decline in PfPR might be that immunity leads to real declines in *h *or real increases in *r*. For the purposes of standardization, the biological reasons for the decline are not relevant.

**Figure 2:**
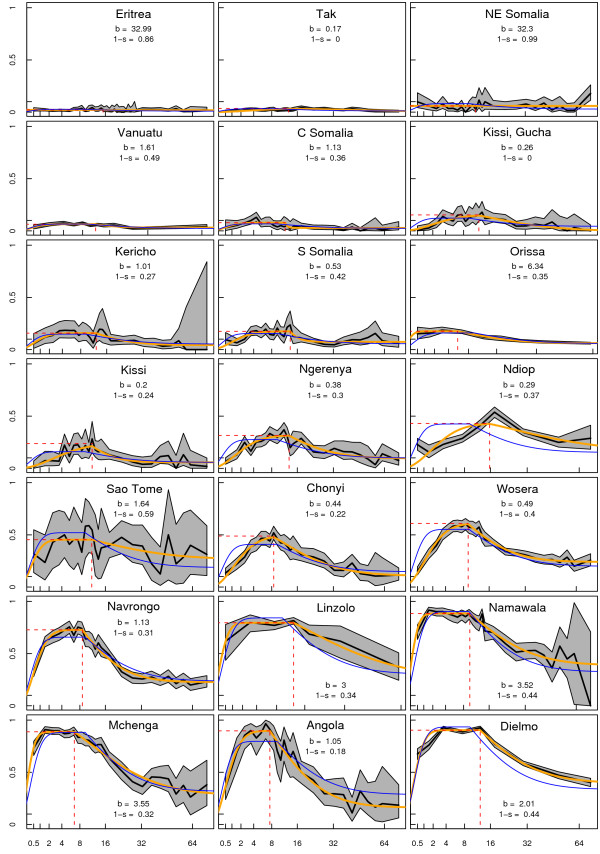
The 21 training sets, ordered by peak PfPR. The PfPR data (black) have been plotted against the root of age in years, along with the confidence limits by the exact test for each proportion (grey). The maximum likelihood best-fit for the modified Pull & Grab model (orange) was also plotted; the plateau (horizontal dashed red line) and the age when PfPR begins to decline (vertical dashed red line) are indicated and the other fitted parameters are reported on each graph. The algorithm (blue) fitted to the data (i.e. four parameters were fixed at their trimmed mean values and the fifth parameter was fitted).

**Figure 3:**
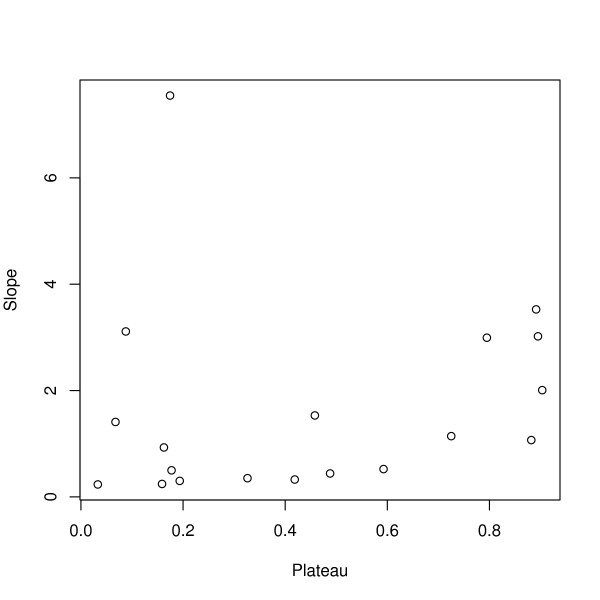
Theory predicts that the slope of PfPR in young children (i.e. *b*) and the PfPR in older children (i.e. the plateau, *P'*) should be correlated. The best-fit parameters describing these two quantities are plotted here. Two extreme values were excluded from this plot. There was no correlation with (p = 0.23) or without (p = 0.56) the extreme values.

The modified Pull & Grab model was fitted to all 21 datasets using maximum likelihood estimation (Figure 2). The algorithm used the average estimates of *b*, *α*, *s*, and *c*. The 21 best-fit parameter values for *α*, *b*, *c*, and *s *were uncorrelated with *P' *(a scatter plot of the slope, *b*, is plotted against the plateau, *P'*, Figure 3). A few of the parameter values were clearly extreme, but a careful examination of the extreme values suggested that they were irrelevant (i.e. changing their values by a factor of 10 did not change the shape of the fitted curve because of the other fitted parameters), so the influence of these extreme but irrelevant values was removed by taking the trimmed means. This generated a family of age-PR curves that depended only on *P'*.

The modified Pull & Grab equations each generate a curve that describes PfPR as a function of age for each value of *P'*. To turn this function into a standardization algorithm, the function need only be inverted. In other words, the algorithm found a value P* such that *PR*_*f*_*[x,y) *= ∫_*x*_^*y*^p(A|P*) S(A) dA/∫_*x*_^*y*^*S(A) dA*. The standardized value was then *PR*_*f*_*[L,U) *= ∫_*L*_^*U*^p(A|P*) S(A) dA/∫_*L*_^*U*^*S(A) dA*.

#### The Garki model

Another parametric method was based on the Garki model [14], using parameter values that were fitted during the Garki project [31]. This model is based on a system of seven coupled difference equations [14]. Here, *P(A) *was computed using an analogue of the Garki equation with seven coupled ordinary differential equations. To convert the parameter values, *-log(1-x)*, was used instead of the parameter *x*. Instead of a fixed delay for the pre-patent periods – the pre-patent period was assumed to be distributed exponentially, but with the same mean time as in the Garki model (i.e. *1/N*). The differences between the ordinary differential equation and difference equations are negligible and are available upon request. Births and deaths were ignored (i.e. δ = 0) because the quantity of interest was prevalence in the survivors; the initial value of x_1 _was set to 1, and all other variables were initially set to 0. The equations were:

dx_1_/dA = R_1 _y_2 _- h x_1_

dx_2_/dA = h x_1 _- x_2_/N

dy_1_/dA = x_2_/N - α_1 _y_1_

dy_2_/dA = α_1 _y_1 _- (α_2 _+ R_1_) y_2_

dx_3_/dA = R_2 _y_3 _- h x_3_

dx_4_/dA = h x_3 _- x_4_/N

dy_3_/dA = α_2 _y_2 _+ x_4_/N - R_2 _y_3_

The function *p(A) = q*_1_*y*_1_*(A) + q*_2_*y*_2_*(A) + q*_3_*y*_3_*(A)*, where *y*_*i*_*(A) *were found by choosing a value for *h *and then numerically solving the system of equations. The algorithm used the age distribution from the training set, *S(A)*. All other parameters except *h *were taken from the original paper (note that the values of R_1 _and R_2 _are fixed by *h *and other parameters). To turn this function into a standardization algorithm, the function again, need only be inverted. In other words, the algorithm found a value h* such that *PR*_*f*_*[x,y) *= ∫_*x*_^*y*^p(A|h*) S(A) dA/∫_*x*_^*y*^*S(A) dA*. The standardized value was then *PR*_*f*_*[L,U) *= ∫_*L*_^*U*^p(A|h*) S(A) dA/∫_*L*_^*U*^*S(A) dA*.

All the routines described were written in R [32], and are available upon request.

## Results

**Figure 4:**
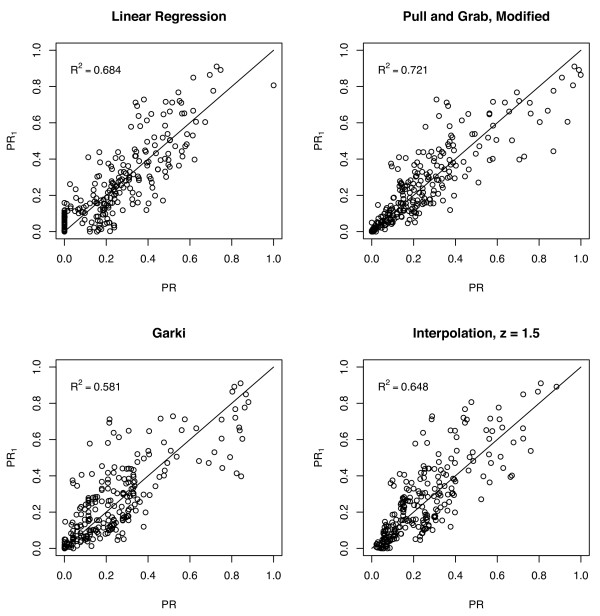
Predicted PfPR plotted against observed PfPR for the four candidate algorithms for the testing set.

Overall, the Pull & Grab-based algorithm ranked highest; it explained 72% of the variance in the testing set, despite being calibrated only on the training set. Linear regression ranked second-best; it explained 68% of the variance. Interpolation (with *z *= *1.5*) ranked third; it explained 64% of the variance. The Garki model explained 58% of the variance. The results are plotted in Figure 4, summarized in Table 1, and discussed in detail in the following sections. Because the algorithm based on Pull & Grab ranked highest, and because it was never fitted to the testing set, the issue of parsimony was not relevant. Had the algorithm based on linear regression ranked highest, the issue of parsimony would have been an important concern.

**Table 1:** Comparing Algorithms

***Data Subset***	***All (n = 242)***	***Random (n = 160/82)***			***L*_1 _≥ *2–10 *≤ *U*_1 _*(n = 87)***	
	***r***^2^	***1***^*st*^	**≥ *2***^*nd*^	**≥ *3***^*rd*^	***r*^2 ^*(n=87)***	***Cat. Err*.**

**Pull & Grab**	0.72	80%	94%	99%	0.71	18%
**Regression**	0.68	17%	70%	95%	0.68	31%
**Interpolation**	0.64	3%	28%	50%	0.57	22%
**Garki**	0.58	0%	0%	5%	0.46	25%
**PR**_2_	0.05	0%	0%	0%	0.21	38%

A summary of the skill of the four candidate standardization algorithms. The columns summarize the proportion of variance explained (r^2^), for the full data, the rankings of the four algorithms fitted to 100 random sub-samples of the testing set (n = 160) and tested on the remainder (n = 82), and the r^2 ^and the proportion of categorical errors for the subset of the testing set that was within the standardization range. The table also includes an evaluation of the paired PfPR estimate (PR_2_).

### Linear regression

By itself, PR_2 _explained only 5% of the variance in PR_1 _(i.e. r^2 ^= 0.05). Linear regression using the formula PR_1 _= a + b PR_2 _improved the r^2 ^to 26%. Linear regression with the formula PR_1 _= a + b PR_2 _+ c L_1 _+ d L_2 _+ e U_1 _+ f U_2 _improved the r^2 ^to 68%, the same as with the slightly non-linear version that always returned a value between 0 and 1. All of the coefficients were statistically significant at the 95% level except for c, the coefficient corresponding to L_1_.

### Interpolation

Several different values of *z *were evaluated including *z *= *0.5, 1, 1.5, 2, 3, 4, 6, 10*. Of these values, *z *= *1.5 *minimized the sum of squared errors and explained 65% of the variance.

### Pull & Grab

**Figure 5:**
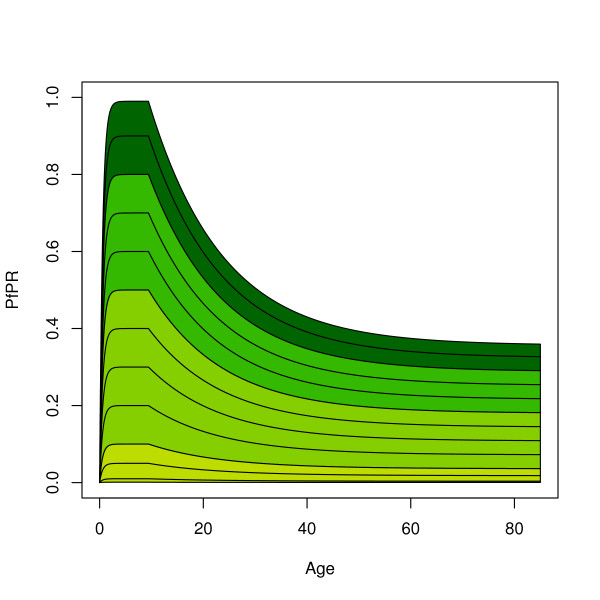
The age-PR relationship as generated by the algorithm. Holoendemic areas are colored dark green, hyper-endemic areas green, meso-endemic areas light green, and hypoendemic olive.

The trimmed mean value for *α *corresponded to a decline in PfPR beginning around age 9.5. The trimmed mean value for *s *corresponded to a decline in sensitivity to approximately 36% of P', but the trimmed mean value of *c *was low and implied that PfPR declines slowly, so that the apparent PfPR is not close to 36% of the PfPR in children between two and ten years of age until late in life. There was substantial variability in *b*, but by the trimmed mean, PfPR was within 90% of P' by age two. The parameter names, interpretations, and values are summarized in Table 2. Some of the individual datasets differed from this pattern, but the majority were within this value by age two. The family of curves described by the algorithm is illustrated in Figure 5. This algorithm explained 72% of the variance.

**Table 2:** Algorithm Parameters

b	The slope, the rate that PfPR approaches the plateau (1.8 y^-1^)
P'	PfPR in older children; the plateau (Variable)
α	The age when PfPR begins to decline (≈ 9.5 years)
1-s	The asymptotic sensitivity of microscopy (1-s ≈ 36%)
c	The rate that PfPR declines with age, after age α (0.07 y^-1^)

The parameter names and interpretations in the algorithm based on the modified Pull & Grab equations.

### Garki

The algorithm based on the Garki model [14] and calibrated during the Garki Project [31] was recently used to generate endemicity maps [20]. The algorithm was therefore included as a candidate for comparison; the relationship between age and PR is qualitatively very similar to the modified Pull & Grab model. It was, therefore, used as-is, without further fitting to the training set or the testing set. It explained 58% of the variance, the lowest of the four algorithms considered.

## Analysis of the sub-sampled data

To evaluate the algorithms further, and to enable the linear regression analysis to be assessed as an algorithm, the testing set was sub-sampled 100 times. The Pull-and-Grab -based algorithm ranked 1^st ^80% of the time, 2^nd ^or better 94% of the time, and 3^rd ^or better 99% of the time. The similar ranking vector for linear regression was 17%, 70% and 95%, for interpolation 3%, 28%, and 50%. The Garki-based algorithm ranked in the top three only 5% of the time.

**Figure 6:**
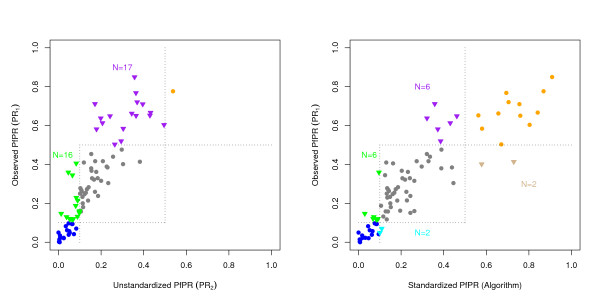
The graphs plot a subset of 87 PfPR estimates (i.e. PR_1_) in the testing set that were already standard (i.e. L_1 _≥ 2 and U_1 _≤ 10) and the predicted PfPR using either the PfPR pair (i.e. PR_2_, left) or the selected standardization algorithm (right). The dashed lines show the cutoffs for the classical categories [18]. The colors highlight properly classified hypoendemic (blue), mesoendemic (grey), and hyperendemic (orange) populations, as well as misclassified populations. Some mesoendemic populations were misclassified as hypoendemic (green) and some hyperendemic populations were misclassified as mesoendemic (orange). After standardizing, a few populations were mistakenly misclassified moved from hypoendemic to mesoendemic (cyan), or from mesoendemic to hyperendemic (tan). In sum, the PfPR would have been misclassified by the paired estimate (i.e. PR_2_) in 33/87 cases (38%). Using the selected Pull & Grab modified algorithm, the standardized values were misclassified in 16/87 cases (18%).

The algorithms were developed and evaluated for standardizing PfPR from any age range to any other; it is possible that they have different skill at standardizing to the PfPR in 2–10 year olds. To further evaluate the algorithm for standardization specifically to the 2–10 year old age classes, 87 datasets were identified in which the age limits were between two and 10 (Figure 6). The PfPR pairs were, again, used as a standard for comparison. When PR_1 _was used instead of its paired PfPR estimate, PR_2_, the categorical description was wrong in 38% of the cases; virtually all of the hyperendemic populations were misclassified as mesoendemic, and many mesoendemic areas were misclassified as hypoendemic. By way of contrast, the standardized PfPR gave the wrong categorical description in 18% of the cases. Standardization reduced the number of mesoendemic populations that would have been classified as hypoendemic, but it also misclassified two populations as mesoendemic that were actually hypoendemic. Standardization correctly classified most, but not all, of the misclassified hyperendemic populations, but two mesoendemic populations were misclassified as hyperendemic. Given the natural scatter in the estimates, errors are inevitable when continuous data are placed into categories. All other candidate algorithms performed more poorly (Table 1).

## Discussion

Four methods were developed and evaluated as potential candidates for an algorithm that is suitable for standardizing crude PfPR data. Of the four methods, the selected algorithm based on a modified version of Pull & Grab's equations [28] was clearly superior to all others. The algorithm explained 72% of the variance in PfPR estimates on a set of independent data, and it was superior to the predictions obtained through linear regression of that data. The mathematical model on which the algorithm was based was biologically motivated and fitted to a set of highly stratified PfPR data that spanned the range of *P. falciparum *transmission intensity. The analysis suggests that the relationship between age and PfPR is fairly consistent across the range of *P. falciparum *transmission intensity and that it is strongly determined by the underlying biology. The algorithm works better than regression because it captures more information about the general patterns in PfPR-age relationships than the 121 averaged PfPR pairs.

While the algorithm permits age-standardization of PfPR for the heterogeneous way of reporting it, this would be unnecessary if minimum standards were followed for reporting PfPR data. Because PfPR varies by age, published estimates of PfPR should always be stratified by age; the 21 training sets listed here have set a reasonable bar, with some important caveats. This analysis suggests that reporting the average PfPR for the 2–10 year old age classes can often be done without loss of information, but the data should first be checked for the typical pattern. For example, PfPR did not reach the plateau until age 5 in some studies. The PfPR begins to decline in older children sometime after age 10 because of blood stage immunity, except possibly at very low PfPR, so PfPR data should be stratified by age in older children and adults. A sensible rule would be to bin by year at least through age 15, and then to bin by at most 5-year age groups after age 20.

For children younger than 2, the PfPR should be reported at a grain that is fine enough to describe changes in PfPR, subject to the social, logistic and ethical concerns of recruiting sufficient infants and young children to the survey. PfPR increases with age in very young children as they acquire their first infections, develop clinical malaria, and then either clear the infections with antimalarial drugs and await reinfection, or recover from symptoms and maintain an asymptomatic infection. Reinfection and clinical malaria continue throughout life, but the frequency and severity of clinical malaria decline as clinical immunity develops [33]. As a consequence, so does the rate at which parasites are cleared by antimalarial drugs. Thus, the expected time to clinical malaria gets longer as children grow older, and this can affect the use of antimalarial drugs. The rate at which children are bitten also increases as their body size increases, and they thus absorb a greater proportion of the bites in the household [34]. Thus, the expectation is that PfPR increases rapidly during these years.

This analysis suggests that after the first few years of life, PfPR settles to a plateau where it remains fairly constant until the onset of adolescence. The plateau reflects the original steady state that was the motivation for using PfPR to index transmission intensity, a population dynamic equilibrium at which parasite clearance is balanced by new infections, and simultaneously not biased by detection issues associated with immunity and microscopy [26,27]. The rise in PfPR with age has been suggested as an alternative index of transmission intensity [35], but this analysis found substantial variability in the slope that was uncorrelated with the PfPR in 2–10 year olds (Figure 3). The discrepancy between the two raises the question of which measure provides a better index of transmission intensity.

For practical and epidemiological reasons, the PfPR in older children provides a good index of transmission intensity, and the age limits from the classical categories are appropriate for standardizing PfPR. There are three justifications for standardizing reported PfPR to 2–10 year olds for use as an index of transmission intensity. First, because PfPR remains relatively constant between the ages of about two and 10, the average is therefore meaningful. Second, because the frequency of clinical malaria declines in older children, the PfPR over these ages may be least influenced by drug treatment [36]. PfPR in older children is also relatively unaffected by immunity, so it can be argued that the PfPR in older children comes closest to reflecting the steady state relationship predicted by mathematical models. It is, therefore, most likely to provide a good index of transmission intensity. Third, it is consistent with the historical approaches to defining endemicity [18].

Using the rise in PfPR in very young children to index transmission has some advantages, but it also has several disadvantages. Although young children are of great interest for malaria control, the slope of PfPR, not the average PfPR in young children, is an index of transmission intensity. From a practical point of view, it is more difficult to measure a slope than an average. The ideal measure of transmission intensity would be the infant conversion rate, characterized by the waiting time to a patent infection in uninfected children. A slightly less direct measure of the force of infection would be any exposure to malaria vs. age, a measure that would be found in an appropriately designed study of seroprevalence [37,38]. PfPR is a distant third because it reflects a balance between exposure and clearance, including natural clearance and radical curative therapy with antimalarial drugs. The frequency of clinical malaria is highest in young children, before they develop functional immunity, so the need for antimalarial drugs is also likely to be correlated with age. The rise in PfPR reflects both exposure and clearance, so it can be quite a volatile measure. For example, the rise in PfPR in young children would dramatically underestimate transmission intensity in populations where effective antimalarial drugs had been used frequently and properly to clear infections. Because gametocytes clear slowly, it has also been argued that measuring gametocyte prevalence directly is a more appropriate measure of exposure, but considerable technical difficulties still limit its wider application [39].

The relationship between age and PR follows a fairly consistent pattern across the natural range of malaria transmission intensity that can be described by a relatively simple, biologically motivated mathematical model. The model, fitted to age-stratified data, accurately describes the rise and fall in PfPR with age, but it does not resolve any questions about the biological causes of this pattern, since it was not designed to do so. The algorithm based on this model can be used to reliably standardize crude estimates, but some important residual variance remains unexplained. This could be due to a variety of factors that were not considered here, including sampling, seasonality, heterogeneous biting, the prevalence of *P. vivax*, and variable use of effective anti-malarial drugs. These will be the focus of future research effort. Despite these caveats, the algorithm explained 72% of the variance, suggesting that age is a dominant source of heterogeneity in PfPR estimates from places with similar transmission intensity. The algorithm thus provides a useful way of standardizing PfPR to 2–10 year olds for comparing studies and ultimately for the mapping of malaria risk.

## Supplementary Material

Additional file 1 The training set. A description of the age-stratified PfPR data from 21 studies. Click here for file

Additional file 2 The testing set. A description of the pairs of PfPR estimates from 121 studies. The estimates were taken from the same population over different age-ranges. Click here for file
