# Supplementary material for: Standardizing estimates of the Plasmodium falciparum parasite rate
Source: Malar J. 2007 Sep 25;6:131. doi: 10.1186/1475-2875-6-131 (PMC2072953; doi:10.1186/1475-2875-6-131)
Supplement: Additional file 1 — The training set. A description of the age-stratified PfPR data from 21 studies. [file 1475-2875-6-131-S1.doc]

**Additional File 1**

A summary of the surveys used to train our models is shown in the table below. These were chosen because they reported PfPR data stratified into several age ranges. Sixteen surveys [1-13] reported PfPR at a one-year age interval from birth to 14 years, and thereafter at five-year intervals up to 65 years of age. Five additional surveys [14-19] reported PfPR with an age stratification of between seven and 12 age groups. In addition, we excluded one survey from Côte d’Ivoire [20] and one from Mozambique [21] despite having age-stratified data due to lack of a sufficiently large age span in the population, in the former (only children less than 15 years sampled) and, in the latter, due to skewness in the sampling (68% of the individuals studied were infants).

**Table.** The training set used for developing the age-standardisation models.

| **Country** | **Area** | **Date** | **Surveys** | **Cohort size** | **Technique** | **PR** | **Citation** |
| --- | --- | --- | --- | --- | --- | --- | --- |
| Angola | Ave Maria & Luvo | 11/2005-11/2005 | 1 | 552 | RDT | 55.47 | [3] |
| Congo | Linzolo | 11/1980-5/1985 | 26 | 1441 | Microscopy | 76.20 | [18] |
| Eritrea | National | 9/2000-11/2000 | 1 | 12661 | RDT | 2.04 | [9] |
| Ghana | Navrongo | 5/2001-11/2001 | 2 | 2286 | Microscopy | 44.91 | [2] |
| India | Orissa | 1998-2000 | 8 | 12107 | Microscopy | 10.55 | [16, 17] |
| Kenya | Chonyi | 7/1999-6/2001 | 6 | 783 | Microscopy | 32.98 | [5, 6] |
| Kenya | Ngerenya | 7/1999-6/2001 | 6 | 819 | Microscopy | 22.73 | [5, 6] |
| Kenya | Kericho | 6/1999-3/2002 | 1 | 220 | Microscopy | 10.91 | [7] |
| Kenya | Gucha | 5/2000-5/2000 | 1 | 2016 | RDT | 12.00 | [13] |
| Kenya | Kisii | 7/2000-7/2000 | 1 | 2778 | RDT | 9.47 | [1] |
| PNG | Wosera | 7/1990-7/1992 | 7 | 10001 | Microscopy | 39.59 | [11] |
| Senegal | Dielmo | 6/1990-9/1990 | 1 | 8539 | Microscopy | 71.95 | [19] |
| Senegal | Ndiop | 1993-1994 | 24 | 3352 | Microscopy | 32.46 | [15] |
| Somalia | South | 1/2005-2/2005 | 1 | 4686 | RDT | 11.93 | [12] |
| Somalia | Central | 1/2005-2/2005 | 1 | 4409 | RDT | 4.99 | [12] |
| Somalia | North East | 5/2005-6/2005 | 1 | 2533 | RDT | 5.96 | [12] |
| ST & Principe | Riboque | 6/1998-3/1998 | 1 | 493 | Microscopy | 39.55 | [4] |
| Tanzania | Michenga | 7/1989-7/1991 | 12 | 2389 | Microscopy | 75.78 | [10] |
| Tanzania | Namawala | 7/1989-7/1991 | 12 | 1138 | Microscopy | 77.62 | [10] |
| Thailand | Tak Province | 9/1998-10/2002 | 3 | 13983 | Microscopy/RDT | 2.30 | [8] |
| Vanuatu | 16 islands | 11/2005-11/2005 | 1 | 552 | RDT | 5.49 | [14] |

**References**

1. Guyatt HL, Corlett SK, Robinson TP, Ochola SA, Snow RW: **Malaria prevention in highland Kenya: indoor residual house-spraying vs. insecticide-treated bednets**. *Trop Med Int Health* 2002, **7**:298-303.

2. Koram KA, Owusu-Agyei S, Fryauff DJ, Anto F, Atuguba F, Hodgson A, Hoffman SL, Nkrumah FK: **Seasonal profiles of malaria infection, anaemia, and bednet use among age groups and communities in northern Ghana**. *Trop Med Int Health* 2003, **8**:793-802.

3. Matsumoto A: **Personal communication**. Communicated to Hay SI. 2006.

4. Muller DA, Charlwood JD, Felger I, Ferreira C, do Rosario V, Smith T: **Prospective risk of morbidity in relation to multiplicity of infection with *Plasmodium falciparum* in São Tomé**. *Acta Trop* 2001, **78**:155-162.

5. Mwangi T: **Clinical epidemiology of malaria under differing levels of transmission**. Weatherall Institute of Molecular Medicine, Open University; 2003.

6. Mwangi TW, Ross A, Snow RW, Marsh K: **Case definitions of clinical malaria under different transmission conditions in Kilifi District, Kenya**. *J Infect Dis* 2005, **191**:1932-1939.

7. Shanks GD, Biomndo K, Guyatt HL, Snow RW: **Travel as a risk factor for uncomplicated *Plasmodium falciparum* malaria in the highlands of western Kenya**. *Trans R Soc Trop Med Hyg* 2005, **99**:71-74.

8. Shoklo Malaria Research Unit (SMRU): **Personal communication**. Communicated to Snow RW. 2005.

9. Sintasath DM, Ghebremeskel T, Lynch M, Kleinau E, Bretas G, Shililu J, Brantly E, Graves PM, Beier JC: **Malaria prevalence and associated risk factors in Eritrea**. *Am J Trop Med Hyg* 2005, **72**:682-687.

10. Smith T, Charlwood JD, Kihonda J, Mwankusye S, Billingsley P, Meuwissen J, Lyimo E, Takken W, Teuscher T, Tanner M: **Absence of seasonal variation in malaria parasitaemia in an area of intense seasonal transmission**. *Acta Trop* 1993, **54**:55-72.

11. Smith T, Hii JL, Genton B, Muller I, Booth M, Gibson N, Narara A, Alpers MP: **Associations of peak shifts in age--prevalence for human malarias with bednet coverage**. *Trans R Soc Trop Med Hyg* 2001, **95**:1-6.

12. WHO - MERLIN: **National Malaria Prevalence Survey - Somalia, January - February 2005, Final Report**. 2005.

13. Zurovac D: **Personal communication**. Communicated to Snow RW. 2006.

14. Kaneko A, Taleo G, Kalkoa M, Yaviong J, Reeve PA, Ganczakowski M, Shirakawa C, Palmer K, Kobayakawa T, Bjorkman A: **Malaria epidemiology, glucose 6-phosphate dehydrogenase deficiency and human settlement in the Vanuatu Archipelago**. *Acta Trop* 1998, **70**:285-302.

15. Rogier C, Trape JF: **Etude de l'acquisition de la prémunition en zones d'holo- et de meso-endémie palustre à Dielmo et à Ndiop (Senegal): résultats préliminaires, 1990-1994**. *Med Trop (Mars)* 1995, **55**:71-76.

16. Sharma SK: **Personal communication**. Communicated to Snow RW. 2005.

17. Sharma SK, Chattopadhyay R, Chakrabarti K, Pati SS, Srivastava VK, Tyagi PK, Mahanty S, Misra SK, Adak T, Das BS, Chitnis CE: **Epidemiology of malaria transmission and development of natural immunity in a malaria-endemic village, San Dulakudar, in Orissa state, India**. *Am J Trop Med Hyg* 2004, **71**:457-465.

18. Trape JF: **Études sur le paludisme dans une zone de mosaique forêt-savane d'Afrique centrale, la région de Brazzaville. II. Densités parasitaires**. *Bull Soc Pathol Exot Filiales* 1987, **80**:520-531.

19. Trape JF, Rogier C, Konate L, Diagne N, Bouganali H, Canque B, Legros F, Badji A, Ndiaye G, Ndiaye P, Brahimi K, Faye O, Druilhe P, Da Silva LP: **The Dielmo project: a longitudinal study of natural malaria infection and the mechanisms of protective immunity in a community living in a holoendemic area of Senegal**. *Am J Trop Med Hyg* 1994, **51**:123-137.

20. Henry MC, Rogier C, Nzeyimana I, Assi SB, Dossou-Yovo J, Audibert M, Mathonnat J, Keundjian A, Akodo E, Teuscher T, Carnevale P: **Inland valley rice production systems and malaria infection and disease in the savannah of Côte d'Ivoire**. *Trop Med Int Health* 2003, **8**:449-458.

21. Mayor A, Saute F, Aponte JJ, Almeda J, Gomez-Olive FX, Dgedge M, Alonso PL: ***Plasmodium falciparum* multiple infections in Mozambique, its relation to other malariological indices and to prospective risk of malaria morbidity**. *Trop Med Int Health* 2003, **8**:3-11.
