# Supplementary material for: Standardizing estimates of the Plasmodium falciparum parasite rate
Source: Malar J. 2007 Sep 25;6:131. doi: 10.1186/1475-2875-6-131 (PMC2072953; doi:10.1186/1475-2875-6-131)
Supplement: Additional file 2 — The testing set. A description of the pairs of PfPR estimates from 121 studies. The estimates were taken from the same population over different age-ranges. [file 1475-2875-6-131-S2.doc]

Additional File 2

The surveys shown in the table below were identified in the MAP database as providing PfPR estimates for two distinct age ranges in the same population. This dataset was appropriate for testing the skill of our models to predict PfPR in a higher age group (Pair 2) using the known PfPR in a lower age group in the same population (Pair 1), and *vice versa*.

**Table.** PfPR surveys utilised as testing set for the models. Pairs 1 and 2 correspond to two PfPR estimates in two different age groups in the same study.

| Country | Area | Date | Pair 1 | | | | | Pair 2 | | | | | Citation |
| --- | --- | --- | --- | --- | --- | --- | --- | --- | --- | --- | --- | --- | --- |
|  |  |  | Low Age | Up Age | Exam | + | PfPR | Low Age | Up Age | Exam | + | PfPR |  |
| Burkina Faso | Barkoundouba & Barkoumbilen | 8/1994-/1996 | 6 | 10·9 | 162 | 140 | 86·42 | 31 | 99 | 189 | 91 | 48·15 | [1] |
| Burkina Faso | Barkoundouba & Barkoumbilen | 8/1994-/1996 | 6 | 11 | 153 | 106 | 69·28 | 31 | 99 | 135 | 21 | 15·56 | [1] |
| Burkina Faso | Barkoundouba & Barkoumbilen | 8/1994-/1996 | 6 | 11 | 230 | 205 | 89·13 | 31 | 99 | 266 | 141 | 53·01 | [1] |
| Burkina Faso | Bourasso | 10/2000-10/2000 | 5 | 14·9 | 476 | 384 | 80·67 | 61 | 99 | 81 | 59 | 72·84 | [2] |
| Burkina Faso | Karangasso | 5/1985-4/1987 | 2 | 10 | 108 | 49 | 45·37 | 20 | 99 | 182 | 28 | 15·38 | [3] |
| Burkina Faso | Karangasso | 5/1985-4/1987 | 2 | 9·9 | 184 | 107 | 58·15 | 20 | 99 | 269 | 48 | 17·84 | [3] |
| Burkina Faso | Tin Edjar | 6/1985-3/1986 | 3 | 9·9 | 180 | 73 | 40·56 | 16 | 99 | 72 | 6 | 8·33 | [4] |
| Cambodia | Bloeng | 7/2002-9/2002 | 2 | 9 | 64 | 6 | 9·38 | 0 | 99 | 238 | 15 | 6·30 | [5] |
| Cambodia | Bornhuk 1 | 7/2002-9/2002 | 2 | 9 | 56 | 12 | 21·43 | 0 | 99 | 209 | 29 | 13·88 | [5] |
| Cambodia | Bornhuk 2 | 7/2002-9/2002 | 0 | 99 | 175 | 31 | 17·71 | 2 | 9 | 47 | 17 | 36·17 | [5] |
| Cambodia | Changra | 7/2002-9/2002 | 2 | 9 | 69 | 45 | 65·22 | 0 | 99 | 184 | 79 | 42·93 | [5] |
| Cambodia | Chet | 7/2002-9/2002 | 2 | 9 | 53 | 15 | 28·30 | 0 | 99 | 191 | 27 | 14·14 | [5] |
| Cambodia | Chrak | 7/2002-9/2002 | 2 | 9 | 57 | 15 | 26·32 | 0 | 99 | 156 | 17 | 10·90 | [5] |
| Cambodia | Des | 7/2002-9/2002 | 0 | 99 | 83 | 6 | 7·23 | 2 | 9 | 24 | 1 | 4·17 | [5] |
| Cambodia | Kachanh 1 | 7/2002-9/2002 | 2 | 9 | 65 | 4 | 6·15 | 0 | 99 | 249 | 10 | 4·02 | [5] |
| Cambodia | Kachanh 2 | 7/2002-9/2002 | 2 | 9 | 52 | 2 | 3·85 | 0 | 99 | 209 | 10 | 4·78 | [5] |
| Cambodia | Kachanh 3 | 7/2002-9/2002 | 2 | 9 | 85 | 7 | 8·24 | 0 | 99 | 321 | 11 | 3·43 | [5] |
| Cambodia | Kachanh 4 | 7/2002-9/2002 | 0 | 99 | 184 | 1 | 0·54 | 2 | 9 | 43 | 0 | 0·00 | [5] |
| Cambodia | Kamenthom | 7/2002-9/2002 | 2 | 9 | 64 | 6 | 9·38 | 0 | 99 | 183 | 12 | 6·56 | [5] |
| Cambodia | Kamentouch | 7/2002-9/2002 | 0 | 99 | 178 | 18 | 10·11 | 2 | 9 | 48 | 12 | 25·00 | [5] |
| Cambodia | Klech | 7/2002-9/2002 | 2 | 9 | 63 | 10 | 15·87 | 0 | 99 | 226 | 22 | 9·73 | [5] |
| Cambodia | Korngthom | 7/2002-9/2002 | 2 | 9 | 120 | 14 | 11·67 | 0 | 99 | 373 | 24 | 6·43 | [5] |
| Cambodia | Laing Av | 7/2002-9/2002 | 0 | 99 | 120 | 16 | 13·33 | 2 | 9 | 33 | 9 | 27·27 | [5] |
| Cambodia | Letouch | 7/2002-9/2002 | 0 | 99 | 165 | 33 | 20·00 | 2 | 9 | 38 | 12 | 31·58 | [5] |
| Cambodia | Lom | 7/2002-9/2002 | 2 | 9 | 89 | 2 | 2·25 | 0 | 99 | 206 | 5 | 2·43 | [5] |
| Cambodia | O Katoeng | 7/2002-9/2002 | 0 | 99 | 152 | 16 | 10·53 | 2 | 9 | 38 | 6 | 15·79 | [5] |
| Cambodia | Pahoy | 7/2002-9/2002 | 0 | 99 | 105 | 18 | 17·14 | 2 | 9 | 27 | 7 | 25·93 | [5] |
| Cambodia | Paor | 7/2002-9/2002 | 2 | 9 | 85 | 28 | 32·94 | 0 | 99 | 261 | 48 | 18·39 | [5] |
| Cambodia | Patang | 7/2002-9/2002 | 2 | 9 | 55 | 14 | 25·45 | 0 | 99 | 195 | 25 | 12·82 | [5] |
| Cambodia | Patoeng | 7/2002-9/2002 | 0 | 99 | 96 | 20 | 20·83 | 2 | 9 | 24 | 10 | 41·67 | [5] |
| Cambodia | Payang | 7/2002-9/2002 | 0 | 99 | 128 | 38 | 29·69 | 2 | 9 | 42 | 20 | 47·62 | [5] |
| Cambodia | Ping | 7/2002-9/2002 | 0 | 99 | 85 | 25 | 29·41 | 2 | 9 | 25 | 13 | 52·00 | [5] |
| Cambodia | Prak | 7/2002-9/2002 | 2 | 9 | 61 | 11 | 18·03 | 0 | 99 | 173 | 20 | 11·56 | [5] |
| Cambodia | Pril | 7/2002-9/2002 | 2 | 9 | 54 | 15 | 27·78 | 0 | 99 | 195 | 20 | 10·26 | [5] |
| Cambodia | Rack | 7/2002-9/2002 | 2 | 9 | 93 | 23 | 24·73 | 0 | 99 | 294 | 36 | 12·24 | [5] |
| Cambodia | Roy | 7/2002-9/2002 | 2 | 9 | 68 | 25 | 36·76 | 0 | 99 | 226 | 35 | 15·49 | [5] |
| Cambodia | Sakmotrleu | 7/2002-9/2002 | 2 | 9 | 66 | 8 | 12·12 | 0 | 99 | 230 | 12 | 5·22 | [5] |
| Cambodia | Sala | 7/2002-9/2002 | 2 | 9 | 80 | 15 | 18·75 | 0 | 99 | 260 | 21 | 8·08 | [5] |
| Cambodia | Salev | 7/2002-9/2002 | 0 | 99 | 79 | 9 | 11·39 | 2 | 9 | 20 | 4 | 20·00 | [5] |
| Cambodia | Smech | 7/2002-9/2002 | 0 | 99 | 82 | 21 | 25·61 | 2 | 9 | 25 | 11 | 44·00 | [5] |
| Cambodia | Soeng | 7/2002-9/2002 | 2 | 9 | 87 | 28 | 32·18 | 0 | 99 | 312 | 52 | 16·67 | [5] |
| Cambodia | Takokchray | 7/2002-9/2002 | 0 | 99 | 137 | 31 | 22·63 | 2 | 9 | 36 | 14 | 38·89 | [5] |
| Cambodia | Takokporng | 7/2002-9/2002 | 0 | 99 | 113 | 21 | 18·58 | 2 | 9 | 42 | 10 | 23·81 | [5] |
| Cambodia | Takokporngchas | 7/2002-9/2002 | 0 | 99 | 71 | 8 | 11·27 | 2 | 9 | 30 | 7 | 23·33 | [5] |
| Cambodia | Trapangchas (2) | 7/2002-9/2002 | 2 | 9 | 105 | 6 | 5·71 | 0 | 99 | 398 | 20 | 5·03 | [5] |
| Cambodia | Trapangkahan (2) | 7/2002-9/2002 | 2 | 9 | 70 | 1 | 1·43 | 0 | 99 | 246 | 1 | 0·41 | [5] |
| Cambodia | Tung | 7/2002-9/2002 | 2 | 9 | 53 | 7 | 13·21 | 0 | 99 | 135 | 12 | 8·89 | [5] |
| Cambodia | Yasom | 7/2002-9/2002 | 2 | 9 | 52 | 14 | 26·92 | 0 | 99 | 178 | 19 | 10·67 | [5] |
| Cambodia | Yem | 7/2002-9/2002 | 2 | 9 | 79 | 26 | 32·91 | 0 | 99 | 235 | 37 | 15·74 | [5] |
| Cameroon | Bolifamba | 6/2001-5/2002 | 3 | 9·99 | 579 | 386 | 66·67 | 16 | 99 | 1060 | 458 | 43·21 | [6] |
| Cameroon | Ebolakounou | 3/1997-7/1998 | 6 | 10 | 894 | 592 | 66·22 | 15 | 99 | 320 | 110 | 34·38 | [7] |
| Cameroon | Etoa | /1994-/1994 | 1 | 10·9 | 116 | 75 | 64·66 | 16 | 99 | 175 | 51 | 29·14 | [8] |
| Cameroon | Koundou | 3/1997-7/1998 | 6 | 10 | 1570 | 1115 | 71·02 | 15 | 99 | 741 | 293 | 39·54 | [7] |
| Cameroon | Simbok | 11/1996-/1998 | 1 | 10·9 | 83 | 43 | 51·81 | 16 | 99 | 118 | 34 | 28·81 | [8] |
| Cameroon | Simbok | 11/1996-/1998 | 1 | 11 | 156 | 111 | 71·15 | 16 | 99 | 139 | 46 | 33·09 | [8] |
| Cameroon | Simbok | 11/1996-/1998 | 1 | 11 | 95 | 68 | 71·58 | 16 | 99 | 136 | 41 | 30·15 | [8] |
| Cameroon | Yaounde (Essos) | 6/1989-5/1990 | 0 | 15·9 | 96 | 43 | 44·79 | 16 | 99 | 65 | 14 | 21·54 | [9] |
| Cameroon | Yaounde (Essos) | 6/1989-5/1990 | 0 | 16 | 95 | 48 | 50·53 | 16 | 99 | 61 | 20 | 32·79 | [9] |
| Cameroon | Yaounde (Essos) | 6/1989-5/1990 | 0 | 16 | 84 | 12 | 14·29 | 16 | 99 | 60 | 3 | 5·00 | [9] |
| Cameroon | Yaounde (Essos) | 6/1989-5/1990 | 0 | 16 | 72 | 27 | 37·50 | 16 | 99 | 59 | 3 | 5·08 | [9] |
| Cameroon | Younde (Obili) | 10/1989-7/1990 | 0 | 16 | 59 | 13 | 22·03 | 16 | 99 | 50 | 4 | 8·00 | [9] |
| Cameroon | Younde (Obili) | 10/1989-7/1990 | 0 | 15·9 | 50 | 14 | 28·00 | 16 | 99 | 78 | 10 | 12·82 | [9] |
| C· African Rep· | Bossembele | 1/1999-1/1999 | 6 | 10·9 | 109 | 63 | 57·80 | 41 | 99 | 139 | 14 | 10·07 | [10] |
| Colombia | Zabaletas | 6/1994-6/1994 | 5 | 9 | 74 | 12 | 16·22 | 15 | 99 | 127 | 13 | 10·24 | [11] |
| Colombia | Zacarias | 2/1996-2/1996 | 5 | 14 | 88 | 3 | 3·41 | 44 | 96 | 58 | 1 | 1·72 | [12] |
| Ethiopia | Chobo | /1989-/1990 | 5 | 9·9 | 206 | 33 | 16·02 | 50 | 99 | 121 | 12 | 9·92 | [13] |
| Gabon | Bellevue | 4/1992-11/1992 | 2 | 10 | 58 | 24 | 41·38 | 15 | 99 | 89 | 34 | 38·20 | [14] |
| Gabon | Bellevue | 4/1992-11/1992 | 2 | 10 | 65 | 25 | 38·46 | 15 | 99 | 87 | 20 | 22·99 | [14] |
| Gabon | Bellevue | 4/1992-11/1992 | 2 | 9·9 | 69 | 21 | 30·43 | 15 | 99 | 137 | 32 | 23·36 | [14] |
| Gabon | HAS village | 4/1992-11/1992 | 2 | 10 | 126 | 19 | 15·08 | 37 | 99 | 51 | 5 | 9·80 | [14] |
| Gabon | HAS village | 4/1992-11/1992 | 2 | 9·9 | 136 | 16 | 11·76 | 37 | 99 | 75 | 4 | 5·33 | [14] |
| Gabon | Tchad | 4/1992-11/1992 | 2 | 10 | 56 | 43 | 76·79 | 15 | 99 | 55 | 20 | 36·36 | [14] |
| Gabon | Tchad | 4/1992-11/1992 | 2 | 10 | 68 | 49 | 72·06 | 15 | 99 | 65 | 24 | 36·92 | [14] |
| Ghana | Dodowa | 4/1992-8/1992 | 2 | 9 | 212 | 130 | 61·32 | 20 | 99 | 218 | 45 | 20·64 | [15] |
| Ghana | Dodowa | 4/1992-8/1992 | 2 | 9 | 189 | 123 | 65·08 | 20 | 99 | 217 | 79 | 36·41 | [15] |
| Ghana | Prampram | 4/1992-8/1992 | 2 | 9 | 217 | 89 | 41·01 | 0 | 20 | 227 | 27 | 11·89 | [15] |
| Ghana | Prampram | 4/1992-8/1992 | 2 | 9 | 192 | 69 | 35·94 | 20 | 99 | 239 | 11 | 4·60 | [15] |
| Guinea | Silipouty | 10/1998-12/1998 | 5 | 14 | 500 | 197 | 39·40 | 15 | 99 | 1018 | 312 | 30·65 | [16] |
| Haiti | Dessources & Palmiste | 8/1987-8/1987 | 2 | 9 | 57 | 4 | 7·02 | 0 | 99 | 156 | 13 | 8·33 | [17] |
| India | Delhi (Shahbad Daulatpur Dairy Colony) | 9/1996-12/1996 | 2 | 99 | 348 | 90 | 25·86 | 0 | 50 | 1147 | 348 | 30·34 | [18] |
| India | Hamirpur | 9/2000-9/2000 | 5 | 15 | 218 | 132 | 60·55 | 25 | 99 | 84 | 37 | 44·05 | [19] |
| India | Hyderabad (GM Nagar Ward Block 18) | 6/1989-8/1989 | 0 | 14 | 57 | 0 | 0·00 | 14 | 99 | 55 | 0 | 0·00 | [20] |
| India | Lutera | 5/1992-5/1992 | 0 | 15 | 84 | 25 | 29·76 | 1 | 5 | 35 | 10 | 28·57 | [21] |
| India | Tarajulie tea state | 5/1992-5/1992 | 0 | 15 | 552 | 86 | 15·58 | 1 | 5 | 193 | 30 | 15·54 | [21] |
| India | Tengabil & Kalabil | 5/1992-5/1992 | 0 | 15 | 68 | 32 | 47·06 | 1 | 5 | 26 | 14 | 53·85 | [21] |
| India | Vijaypur-Najrapur | /1996-/1996 | 0 | 10 | 78 | 31 | 39·74 | 11 | 99 | 169 | 74 | 43·79 | [22] |
| Indonesia | Batu Tajam I, Batu Tajam Ii, Marau Sinar Bulan, Sei Melayu | 7/1996-8/1996 | 2 | 10 | 223 | 5 | 2·24 | 40 | 99 | 134 | 1 | 0·75 | [23] |
| Indonesia | Gag (island) | 4/1997-6/1997 | 2 | 10 | 149 | 32 | 21·48 | 40 | 99 | 78 | 7 | 8·97 | [24] |
| Indonesia | Oksibil Valley (4 villages) | /1990-/1991 | 0 | 10 | 157 | 38 | 24·20 | 11 | 99 | 253 | 30 | 11·86 | [25] |
| Indonesia | Satuan Permukiman | /1996-/1996 | 6 | 12 | 97 | 43 | 44·33 | 20 | 58 | 146 | 64 | 43·84 | [26] |
| Kenya | Kanyawegi sublocation | /2003-/2003 | 1 | 14·9 | 134 | 122 | 91·04 | 41 | 99 | 51 | 24 | 47·06 | [27] |
| Lao People's Democratic Republic | Ban Bouama | 3/1990-3/1990 | 0 | 99 | 144 | 10 | 6·94 | 2 | 9 | 25 | 3 | 12·00 | [28] |
| Lao People's Democratic Republic | Tannon | 5/1994-5/1994 | 6 | 10·9 | 170 | 46 | 27·06 | 16 | 99 | 649 | 170 | 26·19 | [29] |
| Madagascar | Ambararata | 11/1997-11/1997 | 2 | 9·9 | 60 | 25 | 41·67 | 15 | 99 | 151 | 26 | 17·22 | [30] |
| Madagascar | Ambohimena | 11/1997-11/1997 | 2 | 9·9 | 87 | 30 | 34·48 | 15 | 99 | 153 | 10 | 6·54 | [30] |
| Madagascar | Fenoarivo | 11/1997-11/1997 | 2 | 9·9 | 61 | 3 | 4·92 | 15 | 99 | 95 | 5 | 5·26 | [30] |
| Madagascar | Ivory | 11/1997-11/1997 | 2 | 9·9 | 205 | 133 | 64·88 | 15 | 99 | 401 | 97 | 24·19 | [30] |
| Madagascar | Ambatolampy | 11/1988-11/1989 | 2 | 9·9 | 884 | 355 | 40·16 | 15 | 99 | 911 | 276 | 30·30 | [31] |
| Madagascar | Tsarafara | 11/1997-11/1997 | 2 | 9·9 | 101 | 59 | 58·42 | 15 | 99 | 204 | 62 | 30·39 | [30] |
| Malaysia | Pos Legap Valley (10 Villages) | /1989-/1989 | 5 | 9 | 68 | 10 | 14·71 | 30 | 99 | 80 | 1 | 1·25 | [32] |
| Malaysia | Pos Piah Resettlement (4 Villages) | /1999-/1999 | 0 | 10 | 126 | 6 | 4·76 | 31 | 72 | 68 | 1 | 1·47 | [33] |
| Mali | Douna | 10/1988-4/1989 | 2 | 9·9 | 69 | 44 | 63·77 | 10 | 99 | 116 | 23 | 19·83 | [34] |
| Mozambique | Matola | 6/2004-6/2004 | 2 | 9·9 | 1339 | 509 | 38·01 | 40 | 99 | 593 | 91 | 15·35 | [35] |
| Pakistan | Karachi (Hassan Colony) | /1985-/1985 | 2 | 10 | 141 | 7 | 4·96 | 35 | 99 | 82 | 0 | 0·00 | [36] |
| Papua New Guinea | Amele (several villages) | /1999-/2000 | 1 | 9 | 505 | 173 | 34·26 | 10 | 17 | 555 | 225 | 40·54 | [37] |
| Papua New Guinea | Bukask | 5/1986-3/1987 | 5 | 9·9 | 52 | 37 | 71·15 | 20 | 99 | 111 | 19 | 17·12 | [38] |
| Papua New Guinea | Kiunga | /1987-/1990 | 2 | 9 | 1383 | 317 | 22·92 | 10 | 99 | 588 | 49 | 8·33 | [39] |
| Papua New Guinea | Liksul area (3 villages) | 5/2000-7/2000 | 2 | 9 | 76 | 59 | 77·63 | 10 | 82 | 253 | 136 | 53·75 | [40] |
| Papua New Guinea | Tabubil | /1987-/1990 | 2 | 9 | 1114 | 108 | 9·69 | 10 | 99 | 1699 | 80 | 4·71 | [39] |
| Papua New Guinea | Wosera area (6 villages) | 7/1998-1/1999 | 2 | 9 | 106 | 64 | 60·38 | 10 | 85 | 226 | 112 | 49·56 | [40] |
| Philippines | Morong municipality | 6/1990-6/1990 | 5 | 10 | 2250 | 5 | 0·22 | 40 | 99 | 1297 | 3 | 0·23 | [41] |
| Philippines | Sitio Rubber | 12/1991-12/1992 | 0 | 9 | 74 | 21 | 28·38 | 21 | 99 | 82 | 13 | 15·85 | [42] |
| Senegal | Dakar Centre (12 clusters) | 1/1996-/1997 | 2 | 9·9 | 5365 | 75 | 1·40 | 20 | 99 | 7859 | 33 | 0·42 | [43] |
| Senegal | Pikine | 10/1987-6/1988 | 2 | 10 | 435 | 15 | 3·45 | 40 | 99 | 147 | 1 | 0·68 | [44] |
| Solomon Islands | Aruligo, Babala, Berande, Betagela, Kaio and others | 1/1993-8/1993 | 4 | 9 | 157 | 79 | 50·32 | 40 | 99 | 53 | 14 | 26·42 | [45] |
| Somalia | Sigaale | 9/1985-9/1985 | 1 | 9·9 | 70 | 13 | 18·57 | 15 | 99 | 55 | 3 | 5·45 | [46] |
| Sudan | Ed Dekheinat | 10/1995-6/1996 | 5 | 9·9 | 1008 | 21 | 2·08 | 15 | 99 | 1378 | 34 | 2·47 | [47] |
| Sudan | El Manshia | 10/1995-6/1996 | 5 | 9·9 | 123 | 16 | 13·01 | 15 | 99 | 479 | 18 | 3·76 | [47] |
| Thailand | Maung (various villages) | /2001-/2001 | 0 | 14 | 623 | 5 | 0·80 | 15 | 99 | 4604 | 19 | 0·41 | [48] |
| Uganda | Atopi | 12/1995-12/1995 | 2 | 8·9 | 332 | 282 | 84·94 | 41 | 99 | 196 | 70 | 35·71 | [49] |

1. Modiano D, Petrarca V, Sirima BS, Nebie I, Luoni G, Esposito F, Coluzzi M: **Baseline immunity of the population and impact of insecticide-treated curtains on malaria infection**. *Am J Trop Med Hyg* 1998, **59**:336-340.

2. Stich A, Oster N, Abdel-Aziz IZ, Stieglbauer G, Coulibaly B, Wickert H, McLean J, Kouyate BA, Becher H, Lanzer M: **Malaria in a holoendemic area of Burkina Faso: a cross-sectional study**. *Parasitol Res* 2006, **98**:596-599.

3. Carnevale P, Robert V, Boudin C, Halna JM, Pazart L, Gazin P, Richard A, Mouchet J: **La lutte contre le paludisme par des moustiquaires impregnees de pyrethrinoides au Burkina Faso**. *Bull Soc Pathol Exot Filiales* 1988, **81**:832-846.

4. Gazin P, Robert V, Cot M, Simon J, Halna JM, Darriet F, Legrand D, Carnevale P, Ambroise-Thomas P: **Le paludisme dans l'Oudalan, region sahelienne du Burkina Faso**. *Ann Soc Belg Med Trop* 1988, **68**:255-264.

5. Cox J, White E, Lek S: **Remote sensing as a tool for malaria stratification in Cambodia: a feasibility study in Ratanakiri**. London: London School of Hygiene and Tropical Medicine; 2005.

6. Akenji TN, Ntonifor NN, Kimbi HK, Abongwa EL, Ching JK, Ndukum MB, Anong DN, Nkwescheu A, Songmbe M, Boyo MG, Ndamukong KN, Titanji VP: **The epidemiology of malaria in Bolifamba, a rural community on the eastern slopes of Mount Cameroon: seasonal variation in the parasitological indices of transmission**. *Ann Trop Med Parasitol* 2005, **99**:221-227.

7. Bonnet S, Paul REI, Gouagna C, Safeukui I, Meunier JY, Gounoue R, Boudin C: **Level and dynamics of malaria transmission and morbidity in an equatorial area of South Cameroon**. *Trop Med Int Health* 2002, **7**:249-256.

8. Quakyi IA, Leke RG, Befidi-Mengue R, Tsafack M, Bomba-Nkolo D, Manga L, Tchinda V, Njeungue E, Kouontchou S, Fogako J, Nyonglema P, Harun LT, Djokam R, Sama G, Eno A, Megnekou R, Metenou S, Ndountse L, Same-Ekobo A, Alake G, Meli J, Ngu J, Tietche F, Lohoue J, Mvondo JL, Wansi E, Leke R, Folefack A, Bigoga J, Bomba-Nkolo C, Titanji V, Walker-Abbey A, Hickey MA, Johnson AH, Taylor DW: **The epidemiology of *Plasmodium falciparum* malaria in two Cameroonian villages: Simbok and Etoa**. *Am J Trop Med Hyg* 2000, **63**:222-230.

9. Manga L, Traore O, Cot M, Mooh E, Carnevale P: **Le paludisme dans ls ville de Yaoundé (Cameroun). 3. Etude parsitologique dans deux quartiers centraux**. *Bull Soc Pathol Exot* 1993, **86**:56-61.

10. Nguembi E, Yanza M, Sepou A, Youssouf A, Ngbale R, Vohito M: **Lutte antipaludique en zones rurale et semi urbaine de Centrafrique: rôle des moustiquaires imprégnes.** *Médicine D'Afrique Noire* 2004, **51**:231-235.

11. Gautret P, Barreto M, Mendez F, Zorrilla G, Carrasquilla G: **High prevalence of malaria in a village of the Colombian Pacific coast**. *Mem Inst Oswaldo Cruz* 1995, **90**:559-560.

12. Gonzalez JM, Olano V, Vergara J, Arevalo-Herrera M, Carrasquilla G, Herrera S, Lopez JA: **Unstable, low-level transmission of malaria on the Colombian Pacific Coast**. *Ann Trop Med Parasitol* 1997, **91**:349-358.

13. Mengesha T, Nigatu W, Eshete H, Ishir A, Tomofussa T: **Survey of the malaria humoral immune status of selected communities in Gambella, SW Ethiopia.** *Ethiopian J Health Development* 1998, **12**:97-102.

14. Wildling E, Winkler S, Kremsner PG, Brandts C, Jenne L, Wernsdorfer WH: **Malaria epidemiology in the province of Moyen Ogoov, Gabon**. *Trop Med Parasitol* 1995, **46**:77-82.

15. Afari EA, Appawu M, Dunyo S, Baffoe-Wilmot A, Nkrumah FK: **Malaria infection, morbidity and transmission in two ecological zones Southern Ghana**. *Afr J Health Sci* 1995, **2**:312-315.

16. Balde M, Camara M, Barry A, Sow S, Sidibe C, Lamah O, Lodi O, Camara S, Conde N, Bah H: **Étude de la prévalence du paludisme dans 24 villages de la Guinée.** *Bull Soc Pathol Exot* 2001, **94**:192-194.

17. Duverseau Y, Molez JF, Zevallos-Ipenza A: **Etude ponctuelle de la morbidite palustre a Bellevue**. *Epidemiologie du paludisme e des hemoglobinopathies en Haiti (Recherches en sante publique dans la zone Caraibe).* Port-au-Prince; 1988.

18. Ansari MA, Sharma YD, Roy A, Biswas S, Sharma PK: **Epidemiologic investigations of a malaria outbreak in northern Delhi area**. *J Am Mosq Control Assoc* 2001, **17**:216-220.

19. Shukla RP, Sharma SN, Bhat SK: **Malaria outbreak in Bhojpur PHC of district Moradabad, Uttar Pradesh, India**. *J Commun Dis* 2002, **34**:118-123.

20. Khan MM, Kareem MA, Rao GK: **Laboratory diagnosis of malaria infection and its natural history in an urban pocket of Hyderabad City**. *Indian J Malariol* 1989, **26**:215-218.

21. Dev V: **Malaria survey in Tarajulie tea estate and adjoining hamlets in Sonitpur District, Assam**. *Indian J Malariol* 1996, **33**:21-29.

22. Prakash A, Mohapatra PK, Bhattacharyya DR, Doloi P, Mahanta J: **Changing malaria endemicity--a village based study in Sonitpur, Assam**. *J Commun Dis* 1997, **29**:175-178.

23. Fryauff DJ, Tuti S, Mardi A, Masbar S, Patipelohi R, Leksana B, Kain KC, Bangs MJ, Richie TL, Baird JK: **Chloroquine-resistant *Plasmodium vivax* in transmigration settlements of West Kalimantan, Indonesia**. *Am J Trop Med Hyg* 1998, **59**:513-518.

24. Fryauff DJ, Sumawinata I, Purnomo, Richie TL, Tjitra E, Bangs MJ, Kadir A, Ingkokusumo G: ***In vivo* responses to antimalarials by *Plasmodium falciparum* and *Plasmodium vivax* from isolated Gag Island off northwest Irian Jaya, Indonesia**. *Am J Trop Med Hyg* 1999, **60**:542-546.

25. Anthony RL, Bangs MJ, Hamzah N, Basri H, Purnomo, Subianto B: **Heightened transmission of stable malaria in an isolated population in the highlands of Irian Jaya, Indonesia**. *Am J Trop Med Hyg* 1992, **47**:346-356.

26. Barcus MJ, Krisin, Elyazar IR, Marwoto H, Richie TL, Basri H, Wiady I, Fryauff DJ, Maguire JD, Bangs MJ, Baird JK: **Primary infection by *Plasmodium falciparum* or *P. vivax* in a cohort of Javanese migrants to Indonesian Papua**. *Ann Trop Med Parasitol* 2003, **97**:565-574.

27. Ofulla AV, Moormann AM, Embury PE, Kazura JW, Sumba PO, John CC: **Age-related differences in the detection of *Plasmodium falciparum* infection by PCR and microscopy, in an area of Kenya with holo-endemic malaria**. *Ann Trop Med Parasitol* 2005, **99**:431-435.

28. Pholsena K: **Report on the Public Health Survey of Nam Theun Project**. Vientiane, Laos; 1990.

29. Anothay O, Pongvongsa T: **Childhood malaria in the Lao People's Democratic Republic**. *Bull World Health Organ* 1998, **76 Suppl 1**:29-34.

30. Cot M, Brutus L, Le Goff G, Rajaonarivelo V, Raveloson A: **Lutte contre le paludisme dans le moyen-ouest de Madagascar: comparaison de l'efficacite de la lambda-cyhalothrine et du DDT en aspersions intra-domiciliaires. II--Etude parasitologique et clinique**. *Parasite* 2001, **8**:309-316.

31. Lepers JP, Fontenille D, Rason MD, Raharimalala L, Coulanges P: **Le paludisme en 1989 dans un village des Hauts Plateaux malgaches. Donnees parasitologiques et cliniques obtenues apres l'etude longitudinale d'une population representative de cette region**. *Arch Inst Pasteur Madagascar* 1990, **57**:11-52.

32. Gordon DM, Davis DR, Lee M, Lambros C, Harrison BA, Samuel R, Campbell GH, Jegathesan M, Selvarajan K, Lewis GE, Jr.: **Significance of circumsporozoite-specific antibody in the natural transmission of *Plasmodium falciparum*, *Plasmodium vivax*, and *Plasmodium malariae* in an aboriginal (Orang Asli) population of central peninsula Malaysia**. *Am J Trop Med Hyg* 1991, **45**:49-56.

33. Norhayati M, Rohani AK, Hayati MI, Halimah AS, Sharom MY, Abidin AH, Fatmah MS: **Clinical features of malaria in Orang Asli population in Pos Piah, Malaysia**. *Med J Malaysia* 2001, **56**:271-274.

34. Toure YT, Traore SF, Sankare O, Sow MY, Coulibaly A, Esposito F, Petrarca V: **Perennial transmission of malaria by the *Anopheles gambiae* complex in a north Sudan Savanna area of Mali**. *Med Vet Entomol* 1996, **10**:197-199.

35. MoH Mozambique & Swaziland: **Report on Lubombo Spatial Development Iniative (LSDI)**. 2005.

36. Nalin DR, Mahood F, Rathor H, Muttalib A, Sakai R, Chowdhry MA, Safdar G, ul Haq I, Munir M, Suleiman M, Bashir M, Mujtabe SM: **A point survey of periurban and urban malaria in Karachi**. *J Trop Med Hyg* 1985, **88**:7-15.

37. Imrie H, Fowkes FJ, Michon P, Tavul L, Hume JC, Piper KP, Reeder JC, Day KP: **Haptoglobin levels are associated with haptoglobin genotype and alpha+ -Thalassemia in a malaria-endemic area**. *Am J Trop Med Hyg* 2006, **74**:965-971.

38. Burkot TR, Garner P, Paru R, Dagoro H, Barnes A, McDougall S, Wirtz RA, Campbell G, Spark R: **Effects of untreated bed nets on the transmission of *Plasmodium falciparum*, *P. vivax* and *Wuchereria bancrofti* in Papua New Guinea**. *Trans R Soc Trop Med Hyg* 1990, **84**:773-779.

39. Schuurkamp GJT: **The epidemiology of malaria and filariasis in the Ok Tedi region of Western Province, Papua New Guinea**. Port Moresby: Faculty of Medicine, University of Papua New Guinea; 1992.

40. Mehlotra RK, Kasehagen LJ, Baisor M, Lorry K, Kazura JW, Bockarie MJ, Zimmerman PA: **Malaria infections are randomly distributed in diverse holoendemic areas of Papua New Guinea**. *Am J Trop Med Hyg* 2002, **67**:555-562.

41. Bustos MD, Saul A, Salazar NP, Gomes M: **Profile of Morong, Bataan, an area of low malaria endemicity in the Philippines**. *Acta Trop* 1997, **63**:195-207.

42. Tongol-Rivera P, Kano S, Miguel E, Tongol P, Suzuki M: **Application of seroepidemiology in identification of local foci in a malarious community in Palawan, The Philippines**. *Am J Trop Med Hyg* 1993, **49**:608-612.

43. Diallo S, Konate L, Ndir O, Dieng T, Dieng Y, Bah IB, Faye O, Gaye O: **La paludisme dans le district sanitaire centre de Dakar (Senegal). Donnees entomologiques, parasitologiques et cliniques**. *Sante* 2000, **10**:221-229.

44. Trape JF, Lefebvre-Zante E, Legros F, Ndiaye G, Bouganali H, Druilhe P, Salem G: **Vector density gradients and the epidemiology of urban malaria in Dakar, Senegal**. *Am J Trop Med Hyg* 1992, **47**:181-189.

45. Mizushima Y, Kato H, Ohmae H, Tanaka T, Bobogare A, Ishii A: **Prevalence of malaria and its relationship to anemia, blood glucose levels, and serum somatomedin c (IGF-1) levels in the Solomon Islands**. *Acta Trop* 1994, **58**:207-220.

46. Warsame M, Perlmann H, Ali S, Hagi H, Farah S, Lebbad M, Bjorkman A: **The seroreactivity against Pf155 (RESA) antigen in villagers from a mesoendemic area in Somalia**. *Trop Med Parasitol* 1989, **40**:412-414.

47. El Sayed BB, Arnot DE, Mukhtar MM, Baraka OZ, Dafalla AA, Elnaiem DE, Nugud AH: **A study of the urban malaria transmission problem in Khartoum**. *Acta Trop* 2000, **75**:163-171.

48. Pethleart A, Prajakwong S, Suwonkerd W, Corthong B, Webber R, Curtis C: **Infectious reservoir of *Plasmodium* infection in Mae Hong Son Province, north-west Thailand**. *Malar J* 2004, **3**:34.

49. Egwang TG, Apio B, Riley E, Okello D: ***Plasmodium falciparum* malariometric indices in Apac district, northern Uganda**. *East Afr Med J* 2000, **77**:413-416.
